# Mobile Apps for Teaching Intubation: Scoping Review and Critical Analysis in eLearning

**DOI:** 10.2196/mededu.7919

**Published:** 2017-09-05

**Authors:** Clyde Matava, Anne-Marie Leo, Fahad Alam

**Affiliations:** ^1^ Anesthesia and Pain Medicine Hospital for Sick Children Toronto, ON Canada; ^2^ Department of Anesthesia eLearning and Technological Innovation University of Toronto Toronto, ON Canada; ^3^ Department of Anesthesia University of Toronto Toronto, ON Canada

**Keywords:** anesthesia, apps, eLearning, mLearning, intubation, difficult airway, residents, anesthesiology

## Abstract

**Background:**

Airway management is a core skill in anesthesia ensuring adequate oxygenation and delivery of inhalational agents for the patient.

**Objective:**

The goals of this study were to critically evaluate the quality of airway management apps and target revised Bloom's Taxonomy cognitive levels.

**Methods:**

An electronic search using the keywords “airway” and “airway management” was conducted in May 2015 across the App Store, Google Play, BlackBerry World, and Windows Store. Apps were included in the study if their content was related to airway management. App content and characteristics were extracted into a standard form and evaluated.

**Results:**

A total of 65 apps met the inclusion criteria, and 73% (47/65) of apps were developed by companies or industry. Anesthesiology trainees were the target audience in only 20% (13/65) of apps. Bag mask ventilation and laryngeal mask airways were covered in only 20% (13/65) of apps. Only 2 apps were supported in the scientific literature. For Bloom’s Taxonomy, 37% (24/65) of apps targeted knowledge, 5% (3/65) comprehension, 22% (14/65) application, 28% (18/65) analysis, 9% (6/65) evaluation, and 0% synthesis. Multivariate analysis identified cost of apps, size of apps (MB), and apps targeting trainees and paramedics to be associated with higher levels of cognitive processing of revised Bloom’s Taxonomy.

**Conclusions:**

Apps developed for teaching intubation target lower levels of cognitive processing and are largely not validated by research. Cost, app size, and targeted user are associated with higher cognitive levels. Trainees and all users should be aware of the paucity of the published evidence behind the efficacy of some of these apps.

## Introduction

Airway management is a core skill in medicine, important for the maintenance of adequate gas-exchange while enabling the delivery of inhalational medications. Various methods have been used to teach airway management including didactic lectures, seminars, simulation techniques, and workshops. Teaching modalities that are based on cognitive learning theory, mental practice, and simulation are known to be highly effective for both the acquisition of new knowledge and retention [1,2].

mLearning (learning via a mobile device usually through downloadable apps) may be useful for repeated exposure and just-in-time learning [3-5]. Ensuring the quality of mLearning tools is important, particularly with smartphone ownership by health care workers and app use at an all-time high [6,7]. A number of apps have been developed that teach airway management. However, currently there is limited data on how these tools incorporate teaching theory.

The purpose of this study was to (1) characterize the current scope of apps used to teach airway management; (2) critically evaluate their content, use of teaching theory, level of cognitive processing targeted, and scientific validation; and (3) identify gaps in the field to further guide airway app development.

## Methods

### Overview

An electronic search was conducted in May 2015 across the 4 major smartphone operating system app stores: iOS (App Store), Android (Google Play), BlackBerry OS (BlackBerry World), and Windows Phone (Windows Store). Each store was searched separately using the terms “airway” and “airway management.” No date of app publication was used to restrict search results.

### Selection of Apps

Apps were included in the study if the goal of the app was to teach airway management. Apps were excluded if they were not patient-related (advertisements, airline industry-related, etc). Two authors (JW, CM) performed app selection independently. All discrepancies regarding selection were resolved through discussion. There was greater than 90% agreement between app store reviewers across all app stores before meeting for consensus agreement. Data from apps were abstracted into a standard Excel spreadsheet (Microsoft Corp). Abstracted data included app name, developer, country of origin, app description, price, and app size (MB).

### Educational Content and Modalities Assessment

We developed a list of factors to assess educational content and teaching modalities used. This list was based on current literature review and experts who are trained in medical education, eLearning, and app development. We set up a priori criteria for assessing and evaluating apps. Published literature was reviewed and following an iterative process, criteria for data extraction were agreed upon. To assess the comprehensiveness of each app, the following criteria were used: (1) airway topics covered by the app, (2) type of airway devices discussed in the app, and (3) teaching modality used by the app (eg, book, guideline, quiz, journal, video, games, simulation).

### Review of Theoretical Frameworks, Higher Order Cognitive Processing, and Scientific Validation of Apps

App description and its corresponding developer website were used to determine whether a specific theoretical framework had been used to guide app development. To evaluate the highest level of cognitive processing targeted in the app, we used the revised Bloom’s taxonomy [8]. Two reviewers (CM, AL) independently reviewed each app’s description using a standardized revised Bloom’s taxonomy list, and all discrepancies regarding selection were resolved through discussion. Descriptions provided by the app store were examined for evidence of app inclusion in formal scientific research (eg, National Center for Biotechnology Information PubMed and Google Scholar).

### Data Analysis

Descriptive analysis was used to summarize the data. Correlation coefficients between the revised Bloom’s taxonomy and independent variables were determined by Pearson product-moment correlation if independent variables were continuous or by Spearman rank-order correlation if they were categorical or ordinal. Univariate generalized linear model with an identity link and normal distribution was used to identify factors associated with revised Bloom’s taxonomy rankings. Following the univariate analysis, multivariate normal regressions were constructed with revised Bloom’s taxonomy ranking as the dependent variable. Independent variables from significance at the 0.05 level in the univariate analysis were entered simultaneously into the multivariate model. Stepwise selection of covariates was performed with model inclusion and exclusion criteria of *P*=.15 and *P*=.2, respectively. JMP version 12 (SAS Institute Inc) was used to analyze the data.

## Results

### Overview

**Figure 1:**
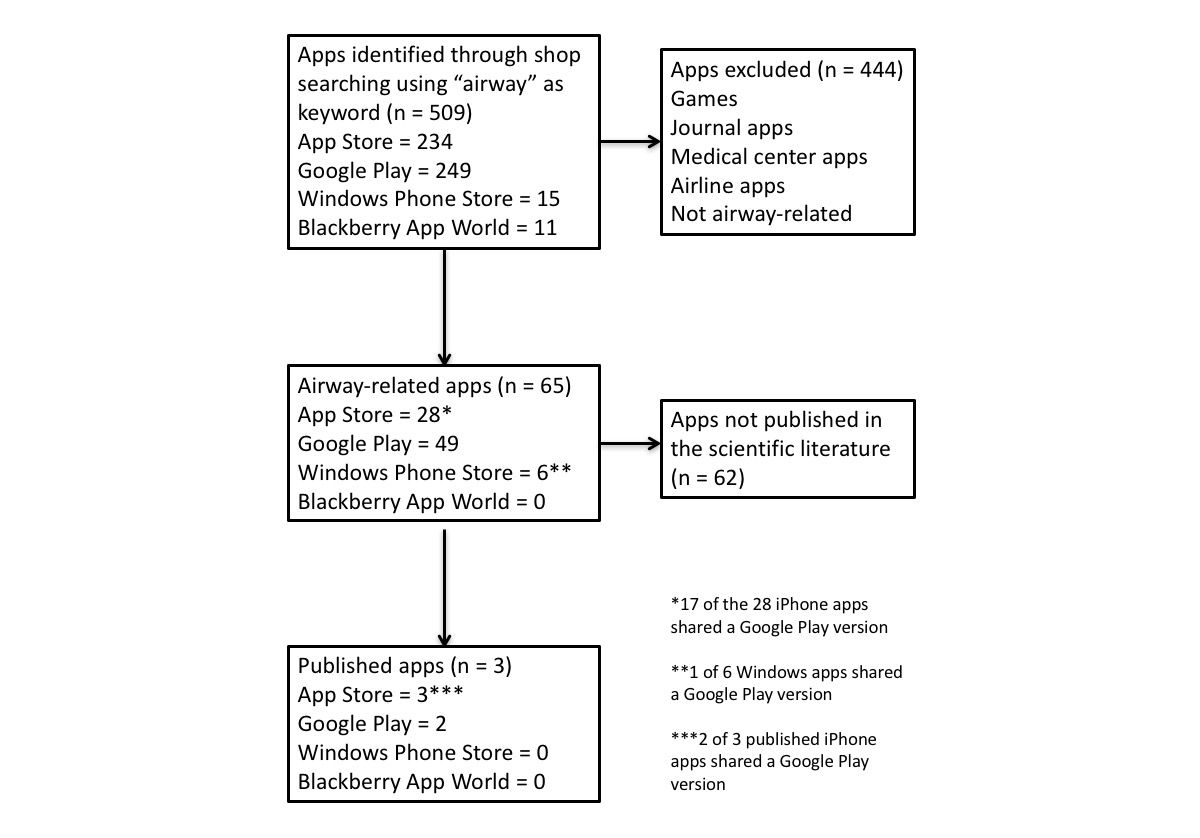
Flow chart of app selection process.

A total of 65 apps were identified for data extraction and analysis (Figure 1). The majority of apps were from Google Play (49/65, 75%), followed by the App Store (28/65, 43%), and the Windows Store (6/65, 9%). Blackberry World did not have any apps relating to airway management.

**Figure 2:**
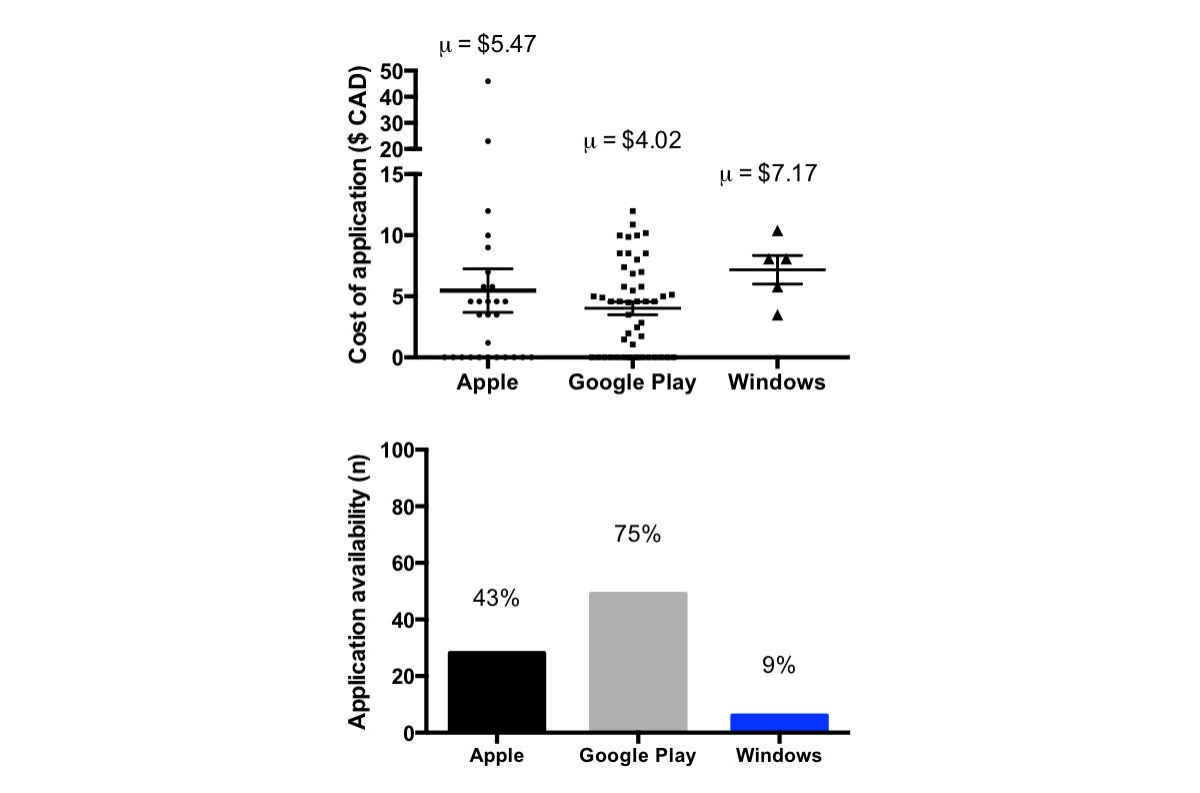
Costs (top) and availability (bottom) of airway management apps.

### Cost

The average cost of an app was Can $4.02 (SD $3.71) from Google Play, Can $5.47 (SD $9.41) from the App Store, and Can $6.79 (SD $2.53) from the Windows Store (Figure 2). A total of 11 apps were free to download from the App Store and 16 from Google Play.

### Developer

**Figure 3:**
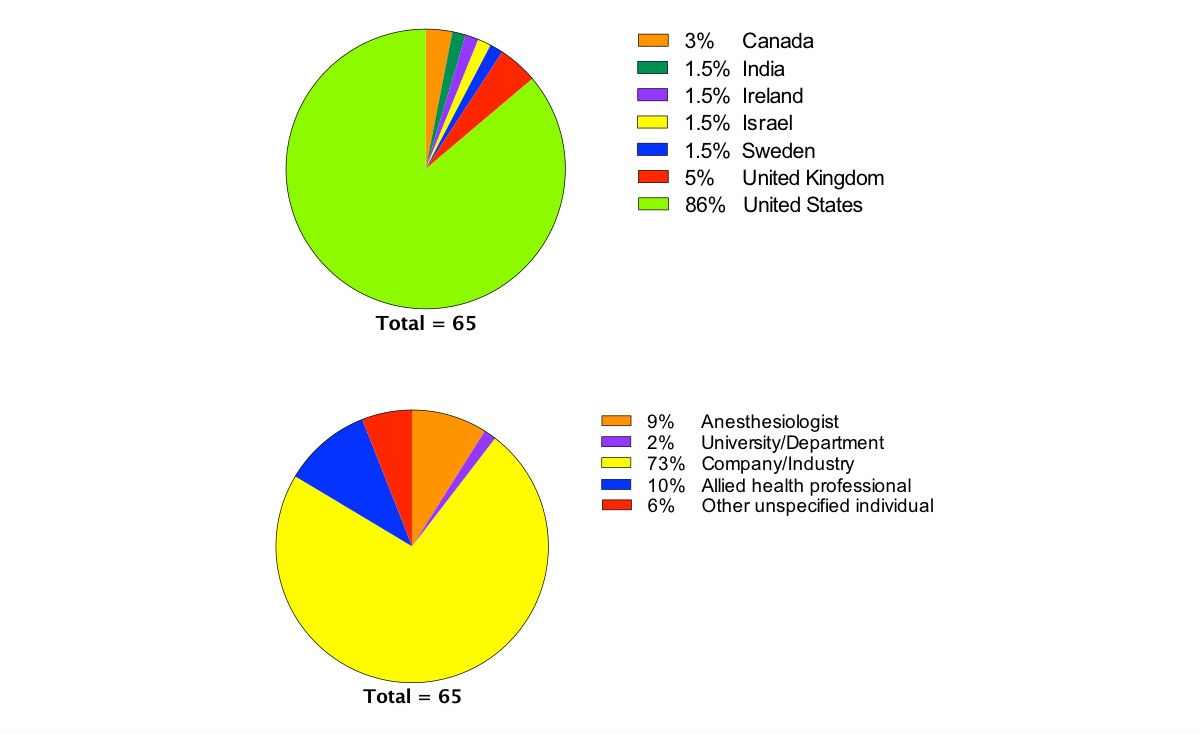
Country of origin (top), identity of app developer (bottom).

The majority of airway apps (47/65, 73%) were developed by companies, meaning that no associated identifiable educational department or authors could be identified; 9% (5/65) of apps were developed by anesthesiologists, 2% (2/65) by university departments, and 90% (58/65) of apps were developed in the United States (86%) (Figure 3). Emergency medical technicians were the most frequent target audience of apps (21/65, 32%) with anesthesiologists the primary target audience in 14% (22/65) (Table 1).

**Table 1:** Intended target audience of airway apps.

Target audience of app	n (%)
Emergency medical technicians	21 (32)
Health care professionals	20 (31)
Paramedics	19 (29)
Trainees	16 (25)
Nurses	14 (22)
Anesthesiologists	9 (14)
Emergency physicians	9 (14)
Critical care physicians	7 (11)
Military medics	4 (6)
Medical students	4 (6)
Family medicine physicians	3 (5)
Lifeguards	2 (3)
Firefighters	2 (3)
Respiratory therapists	1 (2)

### Educational Content

Topics covered in the 65 included apps were quite varied. The most commonly covered topics were oropharyngeal airways (52/65, 80%), difficult airways (33/65, 51%), and endotracheal tubes (24/65, 37%) (Table 2). Bag mask ventilation and laryngeal mask airways were covered by 20% (13/65) of apps. Other topics such as airway equipment, fiberoptic bronchoscopy, cricothyroidotomy, and tracheostomy were each covered in 8% (5/65) of apps.

### Teaching Modality

Apps most often incorporated guidelines as a teaching modality (55/65, 85%). Only 11% (7/65) of apps incorporated interactive or simulation exercises (Table 3).

**Table 2:** Airway management topic covered by app.

Airway management component	n (%)
Oropharyngeal airway	52 (80)
Difficult airway	33 (51)
Endotracheal tube	24 (37)
Bag mask ventilation	13 (20)
Laryngeal mask airway	13 (20)
Rapid sequence induction	10 (15)
Resuscitation protocol	6 (9)
Cricothyroidotomy	5 (8)
Nasal prongs	3 (5)
Oxygen administration	3 (5)
Ventilator settings	3 (5)
Fiberoptic bronchoscopy intubation	2 (3)
Airway equipment	2 (3)
Preoperative assessment	2 (3)
Tracheostomy	1 (2)

**Table 3:** Function of the app.

Function of the app	n (%)
Guidelines	55 (85)
Reference lists	22 (34)
Quizzes with answers	22 (34)
Videos	14 (22)
Games	13 (20)
Books	9 (14)
Algorithms	9 (14)
Interactive/simulation exercises	7 (11)

**Figure 4:**
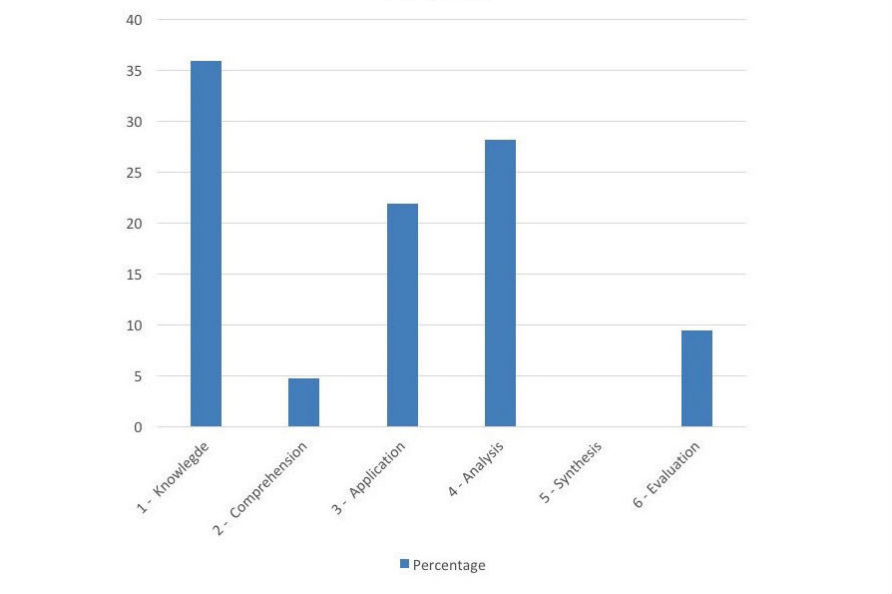
Levels of higher order cognitive processing targeted by identified apps.

### Theoretical Framework

None of the apps included educational theory in their description or on the developer websites.

### Higher Order Cognitive Processing

Figure 4 demonstrates the levels of higher order cognitive processing targeted by included apps. The majority of apps (33/65, 51%) targeted the lowest level of revised Bloom’s taxonomy (Remembering), with 18% (11/65) of apps targeting the higher cognitive processing levels Evaluation and Synthesis.

### Scientific Validation

Only 2 of 65 included apps had associated published literature reporting on the app as a teaching tool. These were the iLarynx and NeoTube apps [9,10]. A third app, LuboCollar, also had published literature [11]; however, the literature associated with LuboCollar focused on the efficacy of the airway device rather than on the educational aspect of the app and therefore was excluded from further analysis.

### Factors Associated With Higher Cognitive Processing

Multivariate analysis demonstrated that the cost of apps, app size (MB), and apps targeting trainees and paramedics were associated with higher levels of cognitive processing (Analysis, Evaluation, Synthesis) in the revised Bloom’s taxonomy (Table 4).

**Table 4:** Factors associated with higher cognitive processing.

Source	Chi-square value	*P* value
Cost of app in Can ($)	4.0	.04
App file size (MB)	3.9	.04
Where is the app available? (App Store)	1.6	.21
Where is the app available? (Windows Store)	0.1	.78
Who developed the app? (company/industry)	0.1	.80
How many versions of app are there in the store? (App Store)	0.0	.97
How many versions of app are there in the store? (Google Play)	0.3	.58
Target audience? (trainees)	14.2	<.001
Target audience? (paramedics)	7.3	<.001

## Discussion

### Principal Findings

This is the first study to examine smartphone apps that teach airway management. We identified 65 apps, covering a range of airway topics that incorporate various teaching modalities. Our findings reveal that none of the apps used any formal advanced educational theory frameworks. We also found that few apps targeted higher order cognitive processing. In addition, a minimal number of apps were validated through scientific research.

The average cost of an app was Can $4.90 (SD $6.76). This suggests that there is scope to increase this charge when one compares this to the average cost of a medical textbook, particularly if quality and efficacy of learning from an app can be demonstrated. The majority of apps were developed by companies (49/65, 76%) without publishing inventor credential information. This contrasts with educational books and courses where an educator’s background is commonly detailed allowing the consumer to make an assessment on the content quality prior to purchase. The authors therefore recommend that developers consider providing author information in app descriptions to better facilitate the selection process for the user and additionally to encourage user investment.

### Leadership in App Development

Our results highlight a gap in the market in relation to developers and target users for airway apps. For example, anesthesiologists are targeted in 14% of available apps and are the developers of only 9%. Anesthesiologists provide expertise in airway management and are well positioned to provide leadership in the development of educational apps pertaining to teaching safe airway management. Our results additionally highlight a paucity of apps targeting anesthesiologists in training, a large population that requires airway management education.

### Education Content and Modalities

While various professions may be involved in the airway management of a patient, the depth of knowledge required can vary between the specialties. Bag mask ventilation was found in only 20% (13/65) of identified apps and, while most apps do not claim to cover all airway topics but rather focus on a particular task, bag mask ventilation is a basic airway skill and as such consumers should be aware that many airway apps do not have broad scope of content. We suggest that consumers be cognizant of app content and target audience in addition to suggesting that app developers offer a trial version for users to evaluate before they decide to purchase.

The modalities used in the identified apps also reflect the levels of cognitive processing targeted, with the majority of apps providing simple guidelines (55/65, 85%), and fewer apps using games (13/65, 20%), quizzes (22/65, 34%), and simulation (7/65, 11%), which provide more complicated teaching modalities.

### Learning Theory and the Revised Bloom’s Taxonomy

In 1956, Bloom’s taxonomy provided a template upon which educational objectives could be built, advancing the processes of curriculum development and student evaluation [8]. In 2001, the revised Bloom’s taxonomy further expanded these learning objective descriptors, developing the cognitive domain and also adding a knowledge domain [8]. When compared to this revised cognitive domain, the majority of apps in this study targeted the lowest level of Bloom’s taxonomy (Remembering) with only 18% (11/65) of apps targeting the 2 highest of the domains. Nevertheless, the authors highlight that this does not necessarily mean that apps classified as targeting the highest domains were of a better quality. For example, some apps allowed for the creation of flashcards and were therefore deemed as targeting the highest level of learning (Synthesis). However, the content of information placed in a flashcard could potentially be inferior to that found in a well-developed, evidence-based, highly researched and referenced app targeting Remembering. These differences may have been better discerned by assessing each app according to the knowledge domain of the revised taxonomy. However, this was not performed in our study and is a limitation to consider. The authors also note that it was often unclear what level of learning was targeted in an app, suggesting that learning objectives may not necessarily have been to the forefront of developers’ minds during app development. This finding should caution users seeking high-quality educational apps in addition to identifying an area for app developers to target. If an app can demonstrate that high levels of learning are targeted, this could translate to higher app earning potential.

Regarding use of theoretical frameworks in the 65 identified apps, we were unable to identify any explicit mention of teaching theory. However, assessment of apps was based on the app descriptor and not on the actual app itself. Nonetheless, it appears that some apps did use multimedia, segmenting, and learner control principles, and it would be interesting to assess apps going forward, looking for use of common eLearning principles, whether intentional or not [9]. It may be possible that apps that include simulation and other tasks that target high-order thinking may require more programming content resulting in larger file sizes (Table 4).

### Proof of App Efficacy

At the time of analysis, only 2 out of the 65 available apps had supporting research published in the scientific literature. The first publication focused on the free to download app iLarynx, which serves as a simulator for fiberoptic intubation using the accelerometer properties of the iPhone and iPad [9]. Using a repeat measures design, 20 trainees, and a power of 87%, the authors demonstrated a difference in time to visualize the carina between students who attended a lecture on fiberoptic intubation and students who received additional iLarynx training. The authors reported that 8 out of 10 participants in the standard training group had at least 1 failed (>120 second) attempt at intubation compared with 2 out of 10 in the iLarynx group (*P*=.01). There were a total of 24 failed attempts in the standard training group, but only 4 in the iLarynx group (*P*<.005). While intubation skill was tested on a manikin, these results suggest that an app using simulation may be a means through which medical technical skills can be taught.

The second available publication examined the effect of a free to download app, NeoTube, teaching neonatal intubation through text, images, and video [10]. The published study was small, examining the effects of the app on 20 trainees. Nonetheless, the findings were promising, demonstrating an improvement in knowledge, skills, and a decrease in duration of intubation attempt (on a manikin) following use of the app.

These 2 publications suggest that apps may be a useful medium through which airway management may be taught. However, the fact that only 2 of 65 studies had associated published literature highlights the current lack of evidence regarding this new mode of learning. The authors recommend that app developers consider formally investigating the educational impact of their apps not only to improve app quality and development but also to add to the marketability of their product [8,9,12]. If developers can prove that their app positively affects learning then developers can consider charging more for their app, and consumers will be more likely to invest [13].

### Limitations

The authors recognize that this study is not without limitations. First, this study identified apps using the keywords “airway” and “airway management.” If an app did not identify these keywords for search purposes, then it will not have been selected for inclusion. Additionally, information on an app was gleaned from the freely available text description of the app and not from examining the actual app itself. However, this search method simulates a typical consumer search and therefore was chosen as the most suitable search method for this study.

A second limitation is that this study did not examine adherence of app contents to current best practice. In view of a recent report that the majority of medical apps don’t adhere to current evidence-based guidelines, this reinforces our recommendations that developers consider employing the appropriate medical and educational experts in order to develop apps for educational purposes [13]. A third study limitation is that only PubMed and Google Scholar were used to identify associated publications. It is possible that if other databases were used, more articles may have been identified. Finally, it is important to mention that mLearning in medical education is a rapidly growing industry and therefore results of this study only pertain to apps available in May 2015 and may not be applicable to the cohort of apps currently available for use.

### Conclusion

Smartphone apps are a new educational medium. As their use develops, it is expected that a more formal approach to app development will be taken. Our study demonstrates that the majority of the currently available apps teaching airway management have been developed by companies, do not cover basic airway management skills, do not target anesthesiologists, and do not target the higher levels of the revised Bloom’s taxonomy cognitive domain. The authors conclude that there is a role for experts in airway management to develop high-quality educational apps in order to serve the purpose of professionals who require attainment of such knowledge and skills. From the available literature examining these apps, there is some evidence to suggest that smartphone apps may be useful educational tools through which airway management skills may be learned. However, apps are a relatively new educational medium and, as such, further research is required in order to investigate the degree to which they may positively augment the learning process. We are only seeing the beginnings of pedagogical concerns regarding the development of apps for educational purposes. The authors highlight this area of research for medical educators going forward.
